# Vanishing Testes: A Literature Review

**DOI:** 10.4274/Jcrpe.728

**Published:** 2012-09-11

**Authors:** Özgur Pirgon, Bumin Nuri Dündar

**Affiliations:** 1 Süleyman Demirel University Faculty of Medicine, Department of Pediatric Endocrinology and Diabetes, Isparta, Turkey; 2 Katip Çelebi University Faculty of Medicine, Department of Pediatric Endocrinology and Diabetes, Izmir, Turkey

**Keywords:** Vanishing testis, nonpalpable testis, Cryptorchidism, testicular regression syndrome

## Abstract

Vanishing testes syndrome is often referred to as testicular regression syndrome (TRS) in the recent medical literature. The most characteristic histological findings are presence of a fibrovascular nodule with associated hemosiderin-laden macrophages and dystrophic calcification. Residual testicular tubules are found in less than 10% of cases, with prevalence being unrelated to age at surgery. Presence of seminiferous tubules and viable germ cells in testicular remnant tissue has been reported in some series. TRS theoretically carries a potential for malignant degeneration in the long term and therefore removal of any remnant is a common practice to eliminate this risk. However, no case series has reported germinal dysplasia or intratubular germ cell neoplasia in any of the specimens taken from these patients.

**Conflict of interest:**None declared.

## INTRODUCTION

Testicular regression syndrome (TRS) or ‘vanishing testis’ is acondition which is considered to be due to the subsequentatrophy and disappearance in fetal life of an initially normal testis(1). The presence of spermatic cord structures is evidence of thepresence of the testis in early intrauterine life. When associatedwith a blind-ending spermatic cord, this entity is named as the‘‘vanishing testis syndrome’’ in the urology literature (2) or TRSin the pathology literature (3), since anatomically it ischaracterized by a rudimentary spermatic cord with absence ofmacroscopically identifiable testicular tissue. This absence of atestis in an otherwise normal 46XY male is usually unilateral andis assumed to be a consequence of intrauterine or perinataltorsion or infarction.

## PREVALENCE

TRS or vanishing testis syndrome may be seen in less than 5% of cryptorchidism cases (4). Cryptorchidism is not an uncommon condition, with about 3% of full-term neonates having an incompletely descended testis at birth - a prevalence decreasing to about 1% by the age of 1 year (5). The testis is nonpalpable in 10% to 20% of cryptorchidism cases, and of these, TRS accounts for 35% to 60% (6,7,8,9,10).

Absence of testis, intra-abdominal testis, inguinal or intra-abdominal vanishing testis are reasons for nonpalpable testis (11,12). Vanishing testis is more common than testicular agenesis in patients with nonpalpable testis (13). It is assumed that 40% of this subpopulation harbors a vanished testis, thus as many as 1 male per 1250 may be affected (14). An 8-year review of cases of nonpalpable testes at the Children’s Hospital of Philadelphia showed that 41% of 447 affected boys (181 patients) had an atrophic remnants or absent testis (15). Therefore, it seems that this syndrome is a common phenomenon; however, the optimal management of this condition remains unclear.

Lou and co-workers reported a series of patients with nonpalpable testes, of which 64% had cryptorchidism. Of these, 22.5% had complete absence of testis, vas deferens and epididymis, 15% had only a blindly-ending vas deferens, and 67.5% had a blindly-ending vas deferens with blood vessels in the inguinal canal (16).

## ETIOLOGY AND PATHOPHYSIOLOGY

Hormonal and mechanical factors have been implicated in the descent of the testes, but a specific factor has not yet been identified for the pathogenesis of undescended or nonpalpable testis. Although the etiology, pathophysiology, and prognosis of TRS and that of the viable undescended testis might be different, TRS is thought to be the result of late antenatal or perinatal vascular thrombosis, torsion, or endocrinopathy (6,17,18,19). Recent studies strongly support a vascular accident and antenatal torsion theory rather than an endocrinopathy. The incompletely descended testis is thought to be more prone to torsion during the fetal and perinatal period (1). This hypothesis is supported by the presence of hemosiderin-laden macrophages in surgically removed specimens (20), consistent with the venous congestion and hemorrhagic infarction secondary to torsion of a structure. On the other hand, trauma to the intrascrotal testis perinatally has been suggested as a risk factor (21). Earlier descent of the left testis may account for the side predilection. Of interest, the oldest patient in the literature was a 26-year-old man who presented with markedly small gonad 11 years after orchidopexy for cryptorchidism and this was removed as an atrophic remnant (4).

Histopathological comparison of the contralateral testis from patients with unilateral vanishing testis and undescended testis was performed by Huff et al (22) and they showed that the patients with an absent testis did not exhibit abnormal, lower germ cell counts suggestive of an endocrinopathic condition.

Testicular regression has been observed in association with genetic abnormality such as microdeletion of the Y chromosome and boys with persistence of Mullerian duct structures have been found to undergo TRS after birth (23). Additionally, early loss of both testes in the antenatal period can result in genital ambiguity. However, an association between genetic factors necessary for normal testicular descent and anorchia/vanishing testis syndrome has not been established (24).

In the vast majority of cases, TRS appears to be a sporadic occurrence, and the patients are otherwise normal with no significant family history. However, there are now several reports of TRS either in association with other defects, including severe mental retardation in chromosomally normal siblings, or occurring in several members of the same family, suggesting a possible genetic basis for the condition in some subjects (25,26). 

## CLINICAL PRESENTATIONS

**Figure 1:**
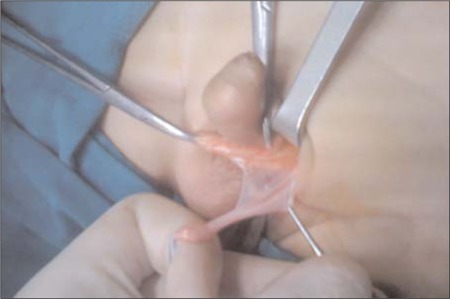
A testicular “remnants” located by the scrotal exploration that was removed from the scrotum with the atretic spermatic cord. Atrophic testicular tissue described as a dense matrix of fibrous connective tissue with widely separated atrophic seminiferous tubules (From the authors’ center)

The clinical manifestations of testicular regression can be associated with the embryological sequence and can be classified into early and late embryonic, and early, mid and late fetal or early neonatal events (18). This disorder may be unilateral or, less commonly, bilateral, with partial or complete absence of testicular tissue and, in the majority of cases, with normal external genitalia (Figure 1). 

**Figure 2:**
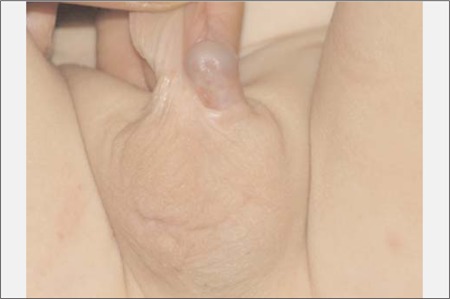
Moderate undervirilization in a 46XY infant presenting with a micropenis with minimal scrotal development and bilateral cryptorchidism (final diagnosis was testicular regression syndrome) (From the authors’ center)

TRS patients usually exhibit a male phenotype. However, although uncommon, the phenotypic spectrum associated with TRS may vary from normal male with unilateral nonpalpable testis through phenotypic male with micropenis, to phenotypic female (27). The phenotype probably depends on the extent and timing of the intrauterine accident in relation to sexual development. These genetically male individuals (46, XY) present with unilateral or bilateral absence of detectable testis structures and Mullerian duct system (3,28,29) (Figure 2). 

Embryonic TRS has been considered to be part of the clinical spectrum of partial 46, XY gonadal dysgenesis (30). Most of the patients with TRS exhibit features of intersex or severe micropenis in association with complete uni- or bilateral regression of testicular tissue. The degree of masculinization of the internal and external genitalia depends on the duration of testicular function prior to its loss. Variable degrees of sexual ambiguity have been reported in familial cases, but the nature of the underlying defect is still not clear. Recently, a novel heterozygous missense mutation (V355M) in SF1 gene was found in one boy with a micropenis and TRS (31).

## DIAGNOSIS

Clinically, a unilateral nonpalpable testis may be associated with TRS, cryptorchidism, retractile testis, or testicular agenesis. Laparoscopy is widely used to distinguish among these conditions while avoiding open abdominal surgery. When, in the setting of a nonpalpable testicle, vas deferens and spermatic vessels are visualized exiting the internal inguinal ring on laparoscopy, a groin exploration may be carried out to identify and remove the testicular tissue remnant associated with a vanished testis (32).

Diagnosis of TRS is based on a clinically nonpalpable testis with a blind-ending spermatic cord located within the retroperitoneum or exiting a closed internal inguinal ring. Usually, a small fibrotic nodule is found at the end of this spermatic cord. Fibrosis, dystrophic calcification, and hemosiderin deposition in association with identifiable testicular/paratesticular structures are also features of TRS (33). 

A number of criteria have been proposed to ascertain the diagnosis of the vanishing testis syndrome. These criteria are: 1) no testis palpated during an examination under anesthesia, 2) visualizing blind-ending spermatic vessels within the retroperitoneum or visualizing spermatic vessels and vas deferens exiting a closed internal inguinal ring. 

## HISTOPATHOLOGICAL CHARACTERISTICS

In gross descriptions of the specimens, a spermatic cord with a small mass of firm, fibrotic tissue at one end, as well as elements of the vas deferens, spermatic artery and venous plexuses have been reported. 

Smith et al (20) in their study have observed that histologically, the distal expansion of most of the specimens was composed of dense fibrovascular tissue in the absence of seminiferous tubules or normal testicular elements; scattered foci of calcification and brown pigment were present. The finding of dystrophic calcification and hemosiderin deposition, in the absence of viable testicular tissue but in the presence of relatively normal spermatic cord elements, supports the hypothesis of remote infarction leading to unilateral and occasionally bilateral anorchia. The young age of the patients and the history of an absent testis from birth are supportive of in utero accident. On the other hand, the above-mentioned histopathological findings are consistent with the hypothesis that in utero torsion may be an underlying factor in the pathogenesis of TRS.

Histopathologic examination has confirmed the presence of germ cells in 11% of testicular remnants. Viable germ cells or seminiferous tubules have been reported in 0 to 16% of excised testicular remnants (7,13,33). Although the precise fate of these remnants is uncertain, the presence of germ cells at least indicates a potential for germ cell–derived neoplasia. In addition, intratubular germ-cell neoplasia in a testicular remnant has been reported in at least one case (6). 

## RISK OF MALIGNANCY

Le Conte first recognized the tumor potential of the undescended testis in 1851 (34). Since then, the increased risk of malignant degeneration of the cryptorchid testis has been estimated to be 20 to 46 times greater than that of the general population (35). However, the location of these testes confers a significantly different degree of risk. Intra-abdominal testes are six times more likely to develop a testis tumor than the undescended testis located in an inguinal position (36). The infrequent finding of seminiferous tubules or other germinal tissue in the surgical specimens of patients with inguinal vanishing testes also supports the contention that the risk of malignant degeneration is quite low in this population. Cendron et al (10) recently presented a histologic evaluation of 25 vanishing testis specimens and noted no identifiable testicular elements in any of these cases.

There is a high risk of malignant transformation in intraabdominal testes especially when associated with external genitalia anomaly and chromosomal anomaly. A testis tumor may develop even after a successful orchiopexy for undescended testis. However, to date, it is not clarified whether vanishing testis and true undescended testis have same etiopathogenesis and risks. The concern in this situation raises the question about the malignant degeneration of residual germinal tissue in the testicular remnants.

Theoretically, presence of testicular tissue in testicular remnants indicates a potential for malignancy in the long term. Histopathological examination of testicular remnants in nonpalpable testes demonstrated presence of seminiferous tubules and viable germ cells in 0 to 16% of the reported series (4,6,10,36). Based on these observations, some authors suggest routine removal of testicular remnant tissue to prevent malignant transformation, but others did not accept this indication because they could not find testicular tissue in testicular remnants. 

## LAPAROSCOPIC EXPLORATION

Nonpalpable testes are a heterogeneous group; their assessment for differential diagnosis needs to include subsets of absent testes, intra-abdominal testes, and testes or testicular remnants located in the inguinal canal that were missed on physical examination. Because of this heterogeneity, laparoscopy has emerged as a useful tool in the localization and assessment of the nonpalpable testis. Various series report success rates of 90% to 95% in the localization of nonpalpable testes with laparoscopy (37,38,39).

While diagnostic laparoscopy is a safe and effective method of investigating patients with a nonpalpable testis, a simpler, less invasive and less costly initial step is to first explore the scrotum. Failure to detect the gonad in the scrotum in a patient with spermatic cord duct structures may occur in two situations: the testis has regressed or the surgeon has not yet localized it. The pathologist may play a critical role in the management of these patients. 95% of the testes are localized at or below the internal inguinal ring, therefore, pathologic evaluation of the spermatic cord and confirmation of the testis as regressed in tissues removed at primary inguinal exploration can reassure the surgeon of the correct diagnosis. In the majority of the cases, this pathologic evaluation can also eliminate the necessity for further surgical intervention or for radiologic evaluation. 

Some authors argue that routine exploration of the groin is required only if laparoscopy reveals an open internal ring with normal vessels, while this exploration is unnecessary if the findings show hypoplastic vessels entering a closed ring (19). Others similarly note that no further action is required if blind-ending vessels are found in the abdomen (6). 

## MANAGEMENT

There is controversy regarding the optimal management of the testicular remnant in cases of TRS. Some urologists recommend surgical exploration, either laparoscopically or via inguinal/scrotal approach, whereas others believe that these procedures are unnecessary (10,33,39). The variable reports of viable germ cell elements found within the testicular remnants may be the reason for the differences in the opinion concerning the proper management. Previous studies have reported an incidence of 0% to 16% of viable germ cell elements within the remnants (4,6,10,36). Some authors recommend surgical fixation of the contralateral testis to prevent torsion in the remaining testicular tissue (18). Inguinal exploration could be postponed until the implantation of testicular prosthesis which is a cosmetic option for patients with unilateral or bilateral loss of testes. Thus, testicular prosthesis implantation can be performed as a first inguinal operation and testicular remnants can be removed during the same session (36).

## CONCLUSION

The histological evaluation of inguinal testicular remnants suggests that these testes became ischemic and atrophied during descent in utero or shortly after birth. The optimal management of the testicular remnants is controversial. Some authors recommend routine excision of remnants, whereas others believe that this is superfluous. A review of the current literature has not identified any report of testicular germ cell tumors which appear to arise from TRS.
